# PADI4 Haplotypes in Association with RA Mexican Patients, a New Prospect for Antigen Modulation

**DOI:** 10.1155/2013/383681

**Published:** 2013-12-22

**Authors:** Maria Guadalupe Zavala-Cerna, Norma Guadalupe Gonzalez-Montoya, Arnulfo Nava, Jorge I. Gamez-Nava, Maria Cristina Moran-Moguel, Roberto Carlos Rosales-Gomez, Susan Andrea Gutierrez-Rubio, Jose Sanchez-Corona, Laura Gonzalez-Lopez, Ingrid Patricia Davalos-Rodriguez, Mario Salazar-Paramo

**Affiliations:** ^1^Programa Internacional del ICB, Facultad de Medicina, Universidad Autónoma de Guadalajara, Guadalajara, JAL, Mexico; ^2^Unidad de Investigación en Epidemiología Clínica, Hospital de Especialidades, Centro Médico Nacional de Occidente del Instituto Mexicano del Seguro Social, Guadalajara, JAL, Mexico; ^3^División de Medicina Molecular del Centro de Investigación Biomédica de Occidente, Instituto Mexicano del Seguro Social, Guadalajara, JAL, Mexico; ^4^Departamento de Medicina Interna-Reumatología del Hospital General Regional No. 110, Instituto Mexicano del Seguro Social, Guadalajara, JAL, Mexico; ^5^División de Genética, CIBO, IMSS and Instituto de Genética Humana, CUCS Universidad de Guadalajara, Guadalajara, JAL, Mexico; ^6^División de Investigación en Salud, UMAE, Hospital de Especialidades, CMNO, IMSS and Departamento de Fisiología, CUCS, Universidad de Guadalajara, Mexico

## Abstract

Peptidyl arginine deiminase IV (PAD 4) is the responsible enzyme for a posttranslational modification called citrullination, originating the antigenic determinant recognized by anti-cyclic citrullinated peptide antibodies (ACPA). Four SNPs (single nucleotide polymorphisms) have been described in *PADI4* gene to form a susceptibility haplotype for rheumatoid arthritis (RA); nevertheless, results in association studies appear contradictory in different populations. The aim of the study was to analyze if the presence of three SNPs in *PADI4* gene susceptibility haplotype (GTG) is associated with ACPA positivity in patients with RA. This was a cross-sectional study that included 86 RA patients and 98 healthy controls. Polymorphisms PADI4_89, PADI4_90, and PADI4_92 in the *PADI4* gene were genotyped. The susceptibility haplotype (GTG) was more frequent in RA patients; interestingly, we found a new haplotype associated with RA with a higher frequency (GTC). There were no associations between polymorphisms and high scores in Spanish HAQ-DI and DAS-28, but we did find an association between RARBIS index and PADI4_89, PADI4_90 polymorphisms. We could not confirm an association between susceptibility haplotype presence and ACPA positivity. Further evidence about proteomic expression of this gene will determine its participation in antigenic generation and autoimmunity.

## 1. Introduction

Rheumatoid arthritis (RA) is an autoimmune disease, characterized by articular inflammation which can lead to joint destruction. RA prevalence is 1% worldwide with considerable variation between ethnic groups, with a higher prevalence in Caucasians compared with Asiatic populations [1, 2]. This disease is more frequent in females (3 : 1) around the fourth decade [3]; some studies suggest that sexual hormones, specifically estrogens, can cause hyperactivity in B and T cell functions [4]. RA represents a disease with risk of function disability due to articular damage as a result of ongoing inflammation, which is irretrievable. In order to limit illness incapability, it is necessary to establish the diagnostic as soon as possible and treat the condition.

Genetic predisposition for this disease is supported by the following findings: (1) first degree relatives of patients with RA have a four to six times greater risk to develop the disease [5]; (2) presence of some HLA-DR molecules (HLA-DRB1*0401 and HLA-DRB1*0404) are genetic factors commonly found in RA, and its presence is associated with a more severe disease [6, 7]. The epidemiological genetic information suggests that the heritability for this disease ranges between 53 and 60%. Linkage disequilibrium studies revealed susceptibility *loci* for RA located within several chromosomes, one consistently implicated is the HLA-DRB1 gene [8]. Since this *locus* represents approximately one third of the total genetic effect, other *loci* should be considered to be part of RA development.

The peptidyl arginine deiminase IV gene denominated *PADI4*, located in 1p36.13, was recently acknowledged as one in association with RA, mainly in Japanese populations [9]. Suzuki and cols. described 17 single nucleotide polymorphisms (SNPs), four of them located in gene coding regions (exons 2–4). They found five haplotypes differing in four polymorphic sites; one denominated the susceptibility haplotype and was associated with RA. The SNPs involved are named PADI4_89, PADI4_90, PADI4_92, and PADI4_104; the first three determine an amino acid change, and the last one is a silent polymorphism [9–11]. In this same study, Suzuki and cols. described that this functional haplotype affected transcript stability, decreasing its degradation four times, and also demonstrated an association between haplotype homozygous individuals and ACPA positivity in patients with RA. In another study, this increase in *PADI4* mRNA stability was confirmed when mononuclear cells of peripheral blood from patients with RA were analyzed [12].

The protein peptidylarginine deiminase (PAD 4) consists of 663 amino acid residues with a 74 kDa molecular weight [13] and is the only isotype out of five described to be expressed in cell nucleus [14]. PAD enzymes have diverse physiologic functions including aggregation of keratin during terminal differentiation in the epidermis [15], involvement in brain development [16], and gene expression regulation by chromatin modeling [14, 17]. PAD 4 enzyme is responsible for a posttranslational modification called citrullination, originating the antigenic determinant recognized by anti-cyclic citrullinated peptide antibodies (ACPA). PAD 4 is a calcium dependant enzyme, an increase in cytosolic Ca^+2^ concentration (2 *μ*M) is needed for citrullinated antigens to appear [13]. Since calcium ions induce conformational changes that create the active site in the *α*/*β* catalytic domain of the enzyme. Intracellular calcium concentrations range from ~200 nM (resting cells) to ~1 *μ*M (activated cells) [18], and calcium concentrations in the cytosol can be increased during apoptosis or necrosis, leading to PAD activation and protein citrullination [19, 20]. Consequences of protein citrullination include protein charge neutralization, change in isoelectric point, ionic interaction breakage, partial protein unfolding, decrease in photolytic degradation, increase in antigenicity, and affinity changes with HLA.

Our interest in the present study comes after the discovery of ACPA, since not only have they shown high sensitivity (80%) and specificity (98%) [21], but they have also demonstrated a positive predictive value [22] in healthy blood donors who developed RA over the years [23] and in patients with undifferentiated arthritis [24]. Several authors have suggested that protein citrullination and autoantibody production are two processes implicated in RA development [24, 25], and nevertheless, the exact mechanism has not been elucidated.

One theory involves the possibility that susceptibility haplotype presence may induce an increase in transcript stability, which would lead to an elevated PAD 4 level and as a consequence citrullination of more epitopes that would break tolerance and induce production of autoantibodies against citrullinated peptides, thus initiating an autoimmune response.

Although evidence exists supporting the presence of *PADI4* susceptibility haplotype in RA Japanese patients [9] and Taiwan patients [26], it could not be extrapolated to other populations [27–29], and it is important to repeat association studies in populations with different ethnic background, in order to find and replicate previous findings related to *PADI4* susceptibility haplotype. The purpose of the present study was to analyze if the presence of three SNPs in *PADI4* gene susceptibility haplotype (GTG) is associated with ACPA positivity in Mexican patients with RA.

## 2. Material and Methods

### 2.1. Patients and Samples

We carried out a cross-sectional study that included 86 patients and 98 healthy subjects from northwestern Mexico who attended to the rheumatology out-patient clinical facilities at “Instituto Mexicano del Seguro Social” in Guadalajara, JAL, Mexico. All patients were classified as RA according to the 1987 ACR classification criteria [30] and fulfilled other inclusion criteria: voluntary acceptance to participate in the study and being able to answer questionnaires. We only included patients with Mestizo ethnicity since two previous generations; patients were not related to each other. Clinical data was obtained from direct interrogatory and physical examination, as well as a chart review in order to identify clinical variables such as disease duration, characteristics of the disease, and therapeutics. Two rheumatologists systematically evaluated the following indexes: DAS-28 [31] to establish severity of disease activity and Spanish HAQ-DI [32] to determine patient disability. We also obtained information from clinical charts in order to evaluate the RARBIS [33] that constitutes a medical records-based index to evaluate RA severity. Patients were included in any functional class according to Steinbrocker Functional Classification, and all of them were receiving treatment; these data was recorded.

Exclusion criteria included patients who had a diagnosis of other rheumatic disease, inability to access patient clinical chart, insufficient amount of sample, or bad quality DNA after extraction.

Healthy controls were blood donors who attended to “Instituto Mexicano del Seguro Social” blood bank and denied having any chronic disease.

### 2.2. Genotyping

DNA from 86 patients with rheumatoid arthritis and 98 healthy subjects was extracted from blood samples using conventional methods [34] and stored frozen at −80°C.

**Figure 1:**
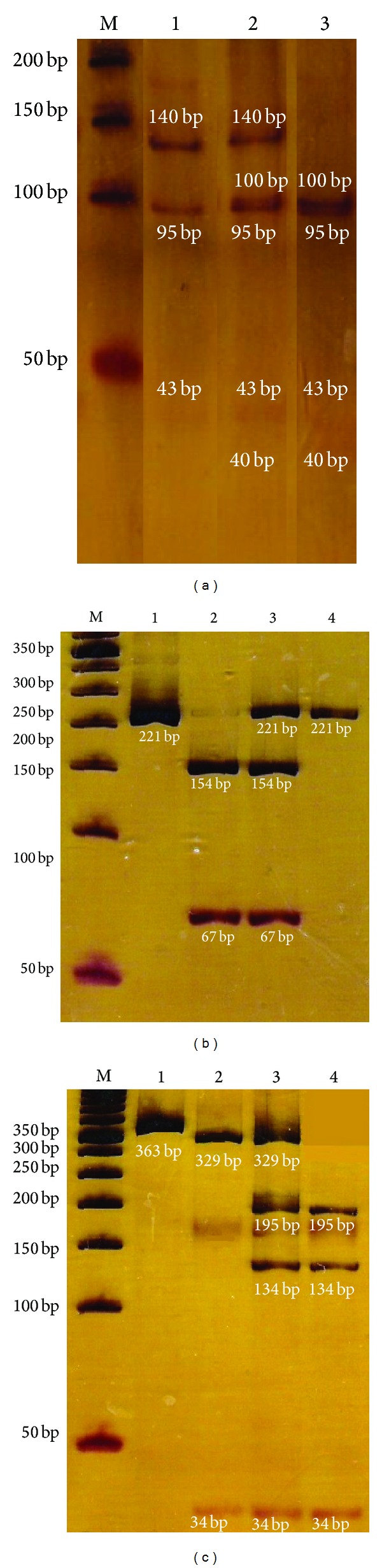
*PADI4* SNPs enzyme digestion. The figure shows digestion of three SNPs in the *PADI4* gene. (a) Shows digestion of PADI4_89, with *HaeIII* enzyme; lane 1 represents the A/A genotype, lane 2 A/G and 3 G/G. (b) Demonstrates PADI4_90 amplification (221 bp) in lane 1 and digested products with *MscI* enzyme in lanes 2 (C/C genotype), 3 (C/T genotype), and 4 (T/T genotype). (c) Shows amplification product of PADI4_92 in lane 1 (363 bp) and restriction products obtained with the enzyme *MspI;* lane 2 corresponds to the G/G genotype, lane 3 G/C, and lane 4 C/C. Visualized in 8% (29 : 1) polyacrylamide gel with silver staining. M: molecular weight marker (50 bp).

Genotyping for polymorphisms PADI4_89, PADI4_90, and PADI4_92 in the* PADI4* gene was determined by three polymerase chain reaction-restriction fragment length polymorphisms (PCR_RFLPs) protocols designed using Oligo 0.4 software, BLAST, and NEBcutter V.2.0.  Table 1 shows primer sequence and obtained products. Each PCR reaction was carried out in 10 *μ*L final volume containing (final concentrations): 1X buffer (200 mM Tris-HCl pH 8.4, 500 mM KCl, and 4 mM MgCl_2_); 5 pmol/mL each of the pair primers according to polymorphism (Table 1); 10 mM each of the four deoxyribonucleoside triphosphates; 1 U of taq DNA polymerase (Invitrogen, Carslbad, CA, USA); and 200 to 300 ng DNA template. The PCR products were visualized by electrophoresis in 8% (29 : 1) polyacrylamide gels at 150 V for 1 h, followed by silver staining. The PADI4_89, PADI4_90, and PADI4_92 genotypes were identified after restriction enzyme digestion with *HaeIII*, *MscI,* and *MspI,* respectively (New England Biolabs, MS, USA), shown in Figure 1.

### 2.3. Haplotypes

Haplotype analysis was done using the software PHASE v 1.0 for haplotype reconstruction, and recombination rate estimation was done using the genotypic data [35].

### 2.4. Anti-Cyclic Citrullinated Peptide (ACPA) Antibody Assay

ACPA (IgG) was measured using a commercially available second generation enzyme-linked immunosorbent assay (ELISA-II), according to the manufacturer instructions (EUROIMMUN, UK). Briefly, test samples, calibrator, and controls are incubated in the respective wells, containing the citrullinated peptide. Antibodies will bind, and nonbound material is removed by washing. Next, peroxidase conjugated anti-human IgG is added to each well. After incubation with substrate solution, the reaction is stopped, and density values were obtained with a spectrophotometer at a wave length of 405/620 nm. Results were expressed in relative units per milliliter (RU/mL), considering positive when the result was >5 RU/mL.

### 2.5. Statistical Analysis

Allelic and genotypic frequencies were determined by gene count. Comparisons between groups for nominal and categorical variables were performed by applying *chi* square test or Fisher exact test as indicated. A *P* value of ≤0.05 was considered significant. Hardy-Weinberg equilibrium was tested.

## 3. Results

### 3.1. Demographics and Clinical

We included 89 patients; all of them attended to rheumatology clinic and were classified as RA according to ACR classification criteria [30]. Three of them were excluded because of poor DNA quality. Mean age was 50 ± 12 years, 100% of the studied subjects were women, mean duration disease was 11 ± 7, years and all of them were receiving treatment, some of them with monotherapy and others combined therapy, and the number and percentages of patients taking a specific drug are listed in Table 2. Results from HAQ-DI, DAS-28, and RARBIS are also shown in Table 2.

### 3.2. Molecular Analysis

In this comparative study of 86 patients and 98 controls, we found a significant association between three exonic SNPs of *PADI4* gene and RA (*P* < 0.05). Genotypic and allelic frequencies are listed on Table 3 confirming previous association studies.

Carriage of PADI4_89 G allele (OR 2.51, 95% CI 1.19–5.32) and PADI4_90 T allele (OR 2.64 95% CI 1.21–5.75) was associated with susceptibility to RA. The PADI4_92 G allele could not be associated with RA (OR 2.08, 95% CI 0.81–5.36).

The three nonsynonymous polymorphisms PADI4_89 (163G/A), PADI4_90 (245T/C), and PADI4_92 (335G/C) constituted mainly eight haplotypes; their sequence and frequencies are represented in Table 4. We found significant association (*P* < 0.0005) of the susceptibility haplotype (Haplotype 1) and RA condition with an OR (95% CI) of 19 (2.4 to 147), and this haplotype was present in 14 patients with RA and only one control. Interestingly, another haplotype was associated significantly with RA (Haplotype 3); this haplotype was present in a higher frequency: 42 patients and 28 controls (*P* = 0.006) OR (95% CI) = 2.4(1.3–4.4). The nonsusceptibility haplotype (Haplotype 2) did not display significant differences.

### 3.3. Antibodies against Cyclic Citrullinated Peptide (ACPA) (IgG)

**Figure 2:**
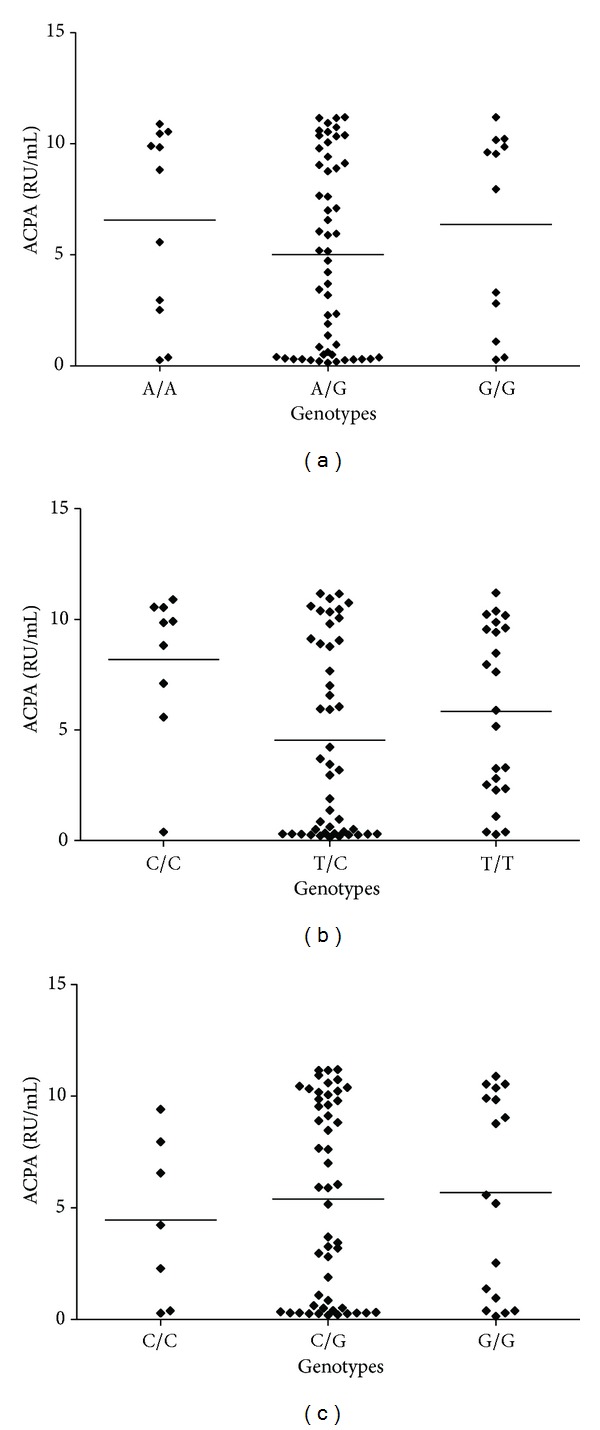
Anti-cyclic citrullinated peptide antibodies (ACPA) titers in the presence of genetic variants in PADI4_89, PADI4_90, and PADI4_92 polymorphisms of *PADI4 *gene.

From 86 patients with RA, 74% had ACPA positivity, and mean  ± SD was 5.98 ± 4.15 RU/mL. We did not find an association between ACPA positivity and the presence of genetic variants in PADI4_89, PADI4_90, and PADI4_92 polymorphisms of *PADI4 *gene; results are shown in Figure 2. We neither found an association between high titers of ACPA and risk allele (data not shown in graph). Fifteen patients had positive smoking, and we searched antibody positivity among them and found eight positive cases with mean ± SD titers of 8.41 ± 2.98 RU/mL.

### 3.4. Clinical Data and *PADI4* Gene Polymorphisms

We searched for possible associations between clinical data, high punctuation score of HAQ-DI DAS-28 and RARBIS indexes with susceptibility haplotype, or the presence of polymorphisms PADI4_89, PADI4_90, and PADI4_92 of *PADI4* gene in RA patients. The only clinical variable significantly associated was the presence of high score in RARBIS index with G allele (susceptibility) of PADI4_89 SNP (*P* = 0.007) and with T/T genotype (homozygous susceptible) of PADI4_90 (*P* = 0.04). For PADI4_92, we did not find any significant association (*P* = 0.84) (data not shown in tables).

## 4. Discussion and Conclusion

During the last decade, RA pathogenesis research has been centered in the idea that multiple cells and molecular mechanisms of the immune system are involved; nevertheless, from this complicated view, a common point emerges: escape from immunological control leading to loss of tolerance with lymphocyte T activation and B cells production of autoantibodies [36]. The self/nonself theory of the immune system fails to explain how autoimmune responses are being generated, since our immune system should be tolerant to self molecules. Matzinger [37] proposed an alternative model that suggested, in addition to antigen presentation, the presence of danger signals that are released after tissue injury and can trigger immune responses [38, 39]. First observations of the relationship between cellular lyses and inflammation go back to inflammasome description; produced spontaneously after cellular membrane disruption [40], inflammasome activation causes the release of IL-1*β*, IL-18, and IL-33 cytokines, which can activate B and T lymphocytes and contribute to the development of inflammatory and autoimmune diseases [41].

Over the years, several paradigms have been broken with respect to RA pathogenesis. In 1997, Weyand and Goronzy [42] proposed a new hypothetical model; this model integrates genetic risk factors to immune inflammatory responses [42]. *PADI4* susceptibility haplotype could be a genetic risk factor, but before *PADI4* gene polymorphisms are considered genetic markers for RA, it was mandatory to replicate the association findings first reported in Asiatic populations; nevertheless, such findings could not be replicated in other populations (Table 5) [27, 28]. In the last years, two meta-analyses have been published pretending to clarify the existence of a true association in different populations [43, 44], since such studies can increase sample size and precision, thus reducing the probability of incising in false positive or false negative results. In Lee and colleagues. study, three Asiatic and six European populations were included [44]; a significant association between RA and *PADI4* polymorphisms was found (PADI4_94, PADI4_104, and PADI4_90).

The present study represents the first one in Mexican population which is the result of genetic admixture of Spanish, Indian, and Black populations, and it should be emphasized that the genetic background introduced from Spaniards includes genes from Romans, Greeks, Visigods, Arabs, and Jews [45]. We should point out that three new PCR-RFLP protocols were designed for the conduction of the present study; we pretend that this approach of association can be replicated in laboratories where equipment for DNA sequencing or RT-PCR is not available, since these have been methodologies described in previous reports [9, 46].

We found that *PADI4* polymorphisms are associated with RA susceptibility, regardless of ACPA titers. This is consistent with results published by Hoppe and Kang et al. in German [11] and Korean [47] populations, respectively. Our findings also show partial concordance with results in Japanese population [9], since PADI4_89 and PADI4_90 were associated with RA in our study. A recent meta-analysis [44] concluded that *PADI4* gene polymorphisms could have a higher susceptibility role in Asiatic populations compared to Caucasians, but soon after that, an association between *PADI4* gene and RA was confirmed in a French population [48]. The lack of replication in different studies could be attributed to (1) false positive results in the primary report due to sample bias, (2) false negative in the replication study due to lack of statistical power, or (3) true genetic heterogeneity exists.

When we analyzed haplotype frequencies, we found that the susceptibility haplotype (GTG) was significantly associated with RA (*P* = 0.0002), but more interestingly, we found a new haplotype (GTC) which was both significantly associated (*P* = 0.006) and more frequent than previous susceptibility haplotype. We believe that since this second haplotype was present in a higher frequency among RA patients, it should be looked forward.

In the present study, our patients with RA had an active disease according to DAS 28 and a significant limitation in functioning according to HAQ-DI. Nevertheless, an exploratory subanalysis did not observe a significant association between the presence of polymorphisms PADI4_89, PADI4_90, and PADI4_92 of *PADI4* gene with high scores in DAS 28 or HAQ-DI. Instead, a G allele of PADI4_89 SNP and T/T genotype of PADI4_90 were significantly associated with higher score in RARBIS. The RARBIS index is based on patient records and could represent a confident tool to measure disease severity, but it is not yet validated in Spanish. It would be desirable that other association studies could further characterize their population with the use of these clinical scores, since most of the association studies do not present clinical data. Furthermore, a deficient clinical characterization of the group of study could contribute to inconsistencies in results.

In our RA patient group, ACPA (IgG) positivity was present in 74%, similar to previous reports [44, 49, 50]. We did not find an association between polymorphisms or haplotype susceptibility of the *PADI4* gene and positivity or elevated titers of ACPA. It should be noted that previous association between ACPA positivity and susceptibility haplotype was described in homozygous subjects to the susceptibility haplotype according to Suzuki et al. [9] and Gandjbakhch et al. [48]. We did not find individuals who were homozygous for the susceptibility haplotype. Additionally, besides IgG isotype ACPA positivity, more recently, the presence of the IgM isotype was demonstrated at different times during the course of RA, indicating that ACPA production is a constant phenomenon during RA evolution [51] and seems to be an ongoing phenomena along disease duration [52]. For this reason, a lack of association between ACPA positivity and susceptibility haplotype presence in our study does not confirm that they are unrelated. Another important finding in ACPA research is the fact that citrullinated proteins can be found in synovium with inflammation caused by several pathologies [53], but the presence of ACPA remains specific for RA patients [10]. As a consequence, it has been suggested that ACPA could participate in RA generation, and there are two possible explanations for their development: the first one considers a high expression of citrullinated antigens which can originate loss of tolerance and as a result ACPA production contributing to inflammation and specific immune responses toward citrullinated antigens. The second theory postulates that RA patients have an abnormal humoral immune response towards citrullinated proteins and they start producing elevated amounts of antibodies against them.

Supporting the first theory is the fact that susceptibility haplotype presence can affect *PADI4* translation, as the increase in mRNA stability has been demonstrated, generating an increase in protein levels and as a consequence a higher occurrence of citrullinated proteins [54]. Citrullination can contribute to the generation of antigens since it originates a change in amino acid charge (amine group is positively charged and citrulline is neutral). This may affect the tertiary or quaternary structure of the protein, allowing protein domains that where otherwise maintained in the internal protein structure to be exposed.

Finally, we did not find associations between *PADI4* gene polymorphisms and clinical variables, except for high punctuation in RARBIS index and presence of susceptibility allele (G) in PADI4_89 or susceptibility homozygous (T/T) in PADI4_90 of *PADI4* gene.

Limitations in this study include sample size since only one gender is represented in the present investigation; it is well known that RA is a condition that affects mainly women, but it would be desirable to increase sample size in order to be able to include male patients and to verify if association with the susceptibility haplotype remains constant, since in our study we were able to find only one HC with the susceptibility haplotype.

In conclusion, we confirm the association between the susceptibility haplotype (GTG) with RA patients, observing also that a new haplotype (GTC) could be associated with RA in Mexican mestizo patients. Further studies including other regions in Mexico and increasing the sample size are required in order to confirm our findings; furthermore, the biggest challenge for association studies is the identification of genetic variants that, combined in haplotypes, can be described as causal effects to RA.

## Figures and Tables

**Table 1:** Genotyping strategies for *PADI4* polymorphism variants detection.

SNP	Primers	*T*_*m*_ (°C)	Band size (bp)	Restriction enzyme	Recognized sequence	Band size after digestion (bp)
PADI4_89	5′-TCTGCTTTCCCATGTGTCTTG-3′ 5′-AGGACAGAGTGTGTGTGGCTG-3′	61	278	*HaeIII *	GGCC	**G** 100, 95, 43, and 40 **A** 140, 95, and 43

PADI4_90	5′-AAATCCACAGGTTCCTCCACA-3′ 5′-CATCACGAGCTCTTCCACAGG-3′	62	221	*MscI *	TGGCA	**T** 154 and 67 **C** 221

PADI4_92	5′-CCCAACTTTGTCTCCCCAGT-3′ 5′-TTGTGGTTCACTGACTAAGGAT-3′	61	363	*MspI *	CCGG	**G** 195, 134, and 34 **C** 329 and 34

SNP: single nucleotide polymorphism, *T*_*m*_: fusion mean temperature, and bp: base pair.

**Table 2:** Demographic and clinical characteristics of RA patients.

Characteristic	RA (*n* = 86)
Age (*x* ± SD)	50 ± 12
Smoking	17%
Years with RA (*x* ± SD)	11 ± 7
Functional class	
** **I	8%
** **II	79%
** **III	11%
** **IV	2%
HAQ-DI (*x* ± SD)	1.22 ± 1.00
DAS 28 (*x* ± SD)	4.4 ± 1.3
RARBIS (*x* ± SD)	6.26 ± 2.70
RF (IgG) +	66%
ACPA (IgM) +	74%
Treatments	*n* (%)
Chloroquine	33 (38)
Sulfasalazine	47 (55)
Methotrexate	65 (76)
Azathioprine	25 (29)
Cyclosporine	1 (1)
Infliximab	2 (2)
Etanercept	10 (11)
Cyclophosphamide	1 (1)
Corticosteroids	72 (80)

HAQ-DI: health assessment questionnaire disability index, DAS: disease activity score, RARBIS: RA medical records-based index of severity, and RF: rheumatoid factor.

**Table 3:** Genotypic and allelic frequencies of PADI4_89, PADI4_90, and PADI4_92 SNPs of *PADI4* gene in controls (HC *n* = 98) and rheumatoid arthritis (RA *n* = 86) patients.

SNPs	Genotypes	RA	HC	Allele	RA	HC	*P *	OR (95% CI)
PADI4_89	A/A	0.148	0.305	A	0.488	0.581	0.040	2.51 (1.19–5.32)
	A/G	0.679	0.551	G	0.512	0.419		
	G/G	0.173	0.144					

PADI4_90	C/C	0.136	0.278	C	0.420	0.562	0.004	2.64 (1.21–5.75)
	C/T	0.568	0.567	T	0.580	0.438		
	T/T	0.296	0.155					

PADI4_92	C/C	0.086	0.165	C	0.438	0.454	0.732	2.08 (0.81–5.36)
	C/G	0.704	0.578	G	0.562	0.546		
	G/G	0.210	0.257					

SNP: single nucleotide polymorphism, HC: healthy controls, RA: rheumatoid arthritis, OR: odds ratio, and 95% CI: 95% confidence interval.

In order to compute OR (95% CI), the following alleles were used as reference: PADI4_89 allele A, PADI4_90 allele C, and PADI4_92 allele C in correspondence with the nonsusceptibility haplotype (ACC).

**Table 4:** Haplotype sequence and frequency of *PADI4* gene SNPs (PADI4_89, PADI4_90, and PADI4_92) in healthy controls and RA patients.

Haplotypes	Condition	Frequencies		*P *	OR (95% CI)
		RA (*n* = 86)	HC (*n* = 98)		
Haplotype 1 susceptibility GTG	Present	14	1	0.0002	18.9 (2.4–146.8)
	Absent	72	97		

Haplotype 2 nonsusceptibility ACC	Present	5	6	1	0.95 (0.3–3.2)
	Absent	81	92		

Haplotype 3 new susceptibility GTC	Present	42	28	0.006	2.4 (1.3–4.4)
	Absent	44	70		

Haplotype 4 ACG	Present	43	39	0.18	1.5 (0.8–2.7)
	Absent	43	59		

Haplotype 5 GCG	Present	1	10	0.011	0.1 (0.01–0.8)
	Absent	85	88		

Haplotype 6 ATC	Present	3	8	0.22	0.4 (0.1–1.6)
	Absent	83	90		

Haplotype 7 ATG	Present	6	6	1	1.1 (0.4–3.7)
	Absent	81	92		

Haplotype 8 GCC	Present	1	4	0.4	0.3 (0.03–2.5)
	Absent	85	94		

HC: healthy controls, RA: rheumatoid arthritis, and SNPs: single nucleotide polymorphisms.

**Table 5:** Association studies for *PADI4* SNPs and rheumatoid arthritis.

SNPs	Country RA/HC	Results	Reference
PADI4_89-105	Japan (830/736)	PADI4_92, 94, 95, 97, 99, 100, 101 and 104 (*P* = 0.0000084–0.00051)	Suzuki et al. 2003 [9]
PADI4_89, 90, 92, 104	UK (839/481)	Susceptibility haplotype more frequent in RA patients (32.3 versus 29.6) without significance (*P* = 0.79)	Barton et al. 2004 [27]
PADI4_92, 96 and 102	France (100 families)	No significant associations	Caponi et al. 2005 [28]
PADI4_92, 94, 97, 99, 100, 103 and 104	England (111/111)	PADI4_100 and 103 (*P* = 0.03). Increase in mRNA expression of *PADI4* in PBM from RA versus C	Harney et al. 2005 [12]
PADI4_94, 102 and 104	Japan (1170/926)	PADI4_94, 102, and 104 (*P* = 0.0008–0.010)	Ikari et al. 2005 [26]
PADI4_94 and 104	Spain (354/498)	No significant associations	Martinez et al. 2005 [29]
PADI4_94	Sweden and North America	Association with RA (*P* = 0.02)	Plenge et al. 2005 [55]
PADI4_89, 90, 92 and 104	Korea (545/392)	Strong association of RA with susceptibility haplotype (*P* = 1.0 × 10^−4^)	Kang et al. 2006 [47]
PADI4_89, 90, 92, 94, 95, 96 and 104	Germany (102/102)	PADI4_89, 90, and 94 (*P* = 0.04)	Hoppe et al. 2006 [11]
PADI4_89 and 90	France (405/275)	Association with RA (*P* = 0.03 and 0.003)	Gandjbakhch et al. 2009 [48]

RA: rheumatoid arthritis, HC: healthy controls, and PBM: peripheral blood mononuclear.
